# Supplementary material for: Lead and cadmium toxicity effects on the Pinus roxburghii seed germination and early seedling growth in different environments
Source: PeerJ. 2025 Aug 6;13:e19593. doi: 10.7717/peerj.19593 (PMC12335238; doi:10.7717/peerj.19593)
Supplement: Supplemental Information 1 [file peerj-13-19593-s001.docx]

Table: Morphological and Physiological traits under heavy metal stress

|  | **Cd field data** | | Table 1 |  |  |  |  |
| --- | --- | --- | --- | --- | --- | --- | --- |
| **treatment** | **Replicate** | **R.L** | **S.L** | **L.L** | **L.N** | **T.F.W** | **T.D.W** |
| C | 1 | 11.9 | 6 | 5.5 | 12 | 0.52 | 0.5 |
| C | 2 | 11.8 | 4.02 | 5.6 | 12 | 0.5 | 0.9 |
| C | 3 | 11.82 | 3.6 | 5.34 | 12 | 0.9 | 0.079 |
| T1 | 1 | 11.2 | 4 | 6.06 | 13 | 0.5 | 0.54 |
| T1 | 2 | 11.3 | 2.21 | 5 | 12 | 0.8 | 0.68 |
| T1 | 3 | 11.4 | 6 | 5.2 | 13 | 0.17 | 0.064 |
| T2 | 1 | 10.69 | 2.9 | 6 | 12 | 0.6 | 0.51 |
| T2 | 2 | 11 | 3.08 | 5.64 | 11 | 0.48 | 0.61 |
| T2 | 3 | 10.8 | 5 | 3.9 | 13 | 0.3 | 0.035 |
| T3 | 1 | 9.8 | 3 | 5 | 12 | 0.5 | 0.51 |
| T3 | 2 | 9.8 | 4.02 | 5.5 | 11 | 0.7 | 0.53 |
| T3 | 3 | 9.08 | 2.7 | 3.06 | 11 | 0.03 | 0.016 |
| T4 | 1 | 8.9 | 1.9 | 4.04 | 10 | 0.9 | 0.131 |
| T4 | 2 | 8.8 | 2.6 | 5 | 10 | 0.2 | 0.32 |
| T4 | 3 | 8.97 | 3.09 | 2.3 | 12 | 0.01 | 0.41 |
| T5 | 1 | 8.9 | 2.1 | 3 | 12 | 0.4 | 0.22 |
| T5 | 2 | 8.9 | 3 | 4.06 | 10 | 0.6 | 0.32 |
| T5 | 3 | 8.48 | 1.38 | 3.2 | 10 | 0.05 | 0.132 |
| T6 | 1 | 6.7 | 2 | 2.33 | 10 | 0.2 | 0.11 |
| T6 | 2 | 6.8 | 1.5 | 4 | 10 | 0.5 | 0.21 |
| T6 | 3 | 6.06 | 0.58 | 3.3 | 12 | 0.11 | 0.121 |
|  |  |  |  |  |  |  |  |
|  |  |  |  |  |  |  |  |
|  |  |  |  |  |  |  |  |
|  |  |  |  |  |  |  |  |
| **Cd lab data** | | Table 3 |  |  |  |  |  |
| **treatment** | **Replicate** | **R.L** | **S.L** | **L.L** | **L.N** | **T.F.W** | **T.D.W** |
| C | 1 | 8.11 | 8 | 7 | 10 | 0.5 | 0.2 |
| C | 2 | 10 | 7.9 | 5.9 | 12 | 0.8 | 0.424 |
| C | 3 | 7.9 | 6.18 | 7.05 | 13 | 0.71 | 0.312 |
| T1 | 1 | 9.12 | 6.91 | 7 | 9 | 0.59 | 0.3 |
| T1 | 2 | 10 | 7 | 6 | 12 | 0.7 | 0.2 |
| T1 | 3 | 8.24 | 9.82 | 8.03 | 13 | 0.78 | 0.553 |
| T2 | 1 | 9.1 | 5 | 7.1 | 11 | 0.6 | 0.2 |
| T2 | 2 | 7.03 | 6.76 | 6.05 | 12 | 0.7 | 0.458 |
| T2 | 3 | 8.05 | 8.52 | 5.6 | 13 | 0.65 | 0.179 |
| T3 | 1 | 8.3 | 4 | 5 | 10 | 0.1 | 0.154 |
| T3 | 2 | 7.34 | 7.21 | 5.73 | 11 | 0.5 | 0.2 |
| T3 | 3 | 7.91 | 8.52 | 6.46 | 13 | 1.26 | 0.408 |
| T4 | 1 | 7.09 | 7 | 4.26 | 10 | 0.2 | 0.2 |
| T4 | 2 | 6.05 | 8.23 | 6 | 11 | 0.4 | 0.2 |
| T4 | 3 | 5.6 | 6.46 | 5.52 | 13 | 1.23 | 0.248 |
| T5 | 1 | 7.51 | 6 | 3 | 10 | 0.5 | 0.1 |
| T5 | 2 | 6.43 | 7.42 | 5.78 | 12 | 0.4 | 0.2 |
| T5 | 3 | 5.95 | 5.84 | 5.56 | 12 | 0.8 | 0.234 |
| T6 | 1 | 6.1 | 4 | 2 | 9 | 0.3 | 0.1 |
| T6 | 2 | 5.03 | 5.23 | 6.32 | 12 | 0.74 | 0.14 |
| T6 | 3 | 4.05 | 6.46 | 4.64 | 13 | 0.52 | 0.2 |
